# Supplementary material for: Effects of Exogenous L-Asparagine on Poplar Biomass Partitioning and Root Morphology
Source: Int J Mol Sci. 2022 Oct 28;23(21):13126. doi: 10.3390/ijms232113126 (PMC9653936; doi:10.3390/ijms232113126)
Supplement: Supplementary file 1 [file ijms-23-13126-s001.zip › ijms-1998806-supplementary.pdf]

Table S1: List of primer sequences used for qPCR

Gene	Forward primer	Reverse primer
UBIC	GATTACCCGGAGAAGCCACC	GTTGTGTGGTGCTGTCATCT
NR	AATGCCGAAGCCTGGTGG	CATGACTCGGCGTCGTTATC
Fd-GOGAT	GTCCAGTCAACGCCACTCTC	CAAGTTTGCTACCTGCGGTT
AS1	TGTTGGAAGTTAGTGCTTCGG	GACAACACACGACTTCAAAGGA
AS2	ACCAAGGCTGCCAGTCAGAATAA	CGTACCCTAACTAAAGCGAACGAAA
AS3	GAGGACCGAAGTACATGCC	CAACAAGGTGCCAACACTACT
ASPG1	GTAATGGAGATAGCCAAATTGGGACT	CCTTTGCCTGTAGCGGAAACTG
ASPG2	GCTGCTAACAGTCCCTTGGTA	ACGTGCTAGTGTCCCTCGTATAA
ASPG3	TGATGCGCTTCCTTCCATGT	ATCCATGACAAGCACCAGCA
GS1.1	ATGGTTGTCTGTCAATTTGTTTGCC	CCAGCAAGAGTTTTATTTAGATTAG
GS1.2	GGAATTGAGTATTGGAAGATGATGG	TATGTTCATAAATGATCAACAGCC
GS1.3	TGGAAACCATAAGAGATCACCACC	GAAGAGGCAATTCTTGTACCAAG
GS2	GGAGCATCACTTGGATCTAGATGG	CAAAACCCAAGAGTAAAAAGGTCC
NiR	CAAGTTGCCGATATTGGGT	AAGTCCTTGCATGGAACAC
CWINV1	TCGTAGACATGGATCCTCGC	TGCTTGTGAATTGCCAGCTT
VI2	CAGTTCAAGATCTGGCCACA	GCAAGCTCGGATGGATAAGC
